# Influence of different isoflurane anesthesia protocols on murine cerebral hemodynamics measured with pseudo‐continuous arterial spin labeling

**DOI:** 10.1002/nbm.4105

**Published:** 2019-06-07

**Authors:** Leon P. Munting, Marc P.P. Derieppe, Ernst Suidgeest, Baudouin Denis de Senneville, Jack A. Wells, Louise van der Weerd

**Affiliations:** ^1^ Department of Radiology Leiden University Medical Center Leiden the Netherlands; ^2^ Department of Human Genetics Leiden University Medical Center Leiden the Netherlands; ^3^ Prinses Máxima Center for Pediatric Oncology University Medical Center Utrecht Utrecht the Netherlands; ^4^ Department of Radiotherapy University Medical Center Utrecht Utrecht the Netherlands; ^5^ Institut de Mathématiques de Bordeaux Université Bordeaux/CNRS UMR 5251/INRIA Bordeaux‐Sud‐Ouest France; ^6^ Division of Medicine, UCL Centre for Advanced Biomedical Imaging University College London London UK

**Keywords:** anesthesia, arterial spin labeling (ASL)‐MRI, brain, hemodynamics, isoflurane, mouse

## Abstract

Arterial spin labeling (ASL)‐MRI can noninvasively map cerebral blood flow (CBF) and cerebrovascular reactivity (CVR), potential biomarkers of cognitive impairment and dementia. Mouse models of disease are frequently used in translational MRI studies, which are commonly performed under anesthesia. Understanding the influence of the specific anesthesia protocol used on the measured parameters is important for accurate interpretation of hemodynamic studies with mice. Isoflurane is a frequently used anesthetic with vasodilative properties. Here, the influence of three distinct isoflurane protocols was studied with pseudo‐continuous ASL in two different mouse strains. The first protocol was a free‐breathing set‐up with medium concentrations, the second a free‐breathing set‐up with low induction and maintenance concentrations, and the third a set‐up with medium concentrations and mechanical ventilation. A protocol with the vasoconstrictive anesthetic medetomidine was used as a comparison. As expected, medium isoflurane anesthesia resulted in significantly higher CBF and lower CVR values than medetomidine (median whole‐brain CBF of 157.7 vs 84.4 mL/100 g/min and CVR of 0.54 vs 51.7% in C57BL/6 J mice). The other two isoflurane protocols lowered the CBF and increased the CVR values compared with medium isoflurane anesthesia, without obvious differences between them (median whole‐brain CBF of 138.9 vs 131.7 mL/100 g/min and CVR of 10.0 vs 9.6%, in C57BL/6 J mice). Furthermore, CVR was shown to be dependent on baseline CBF, regardless of the anesthesia protocol used.

**Abbreviations used**
ASL: arterial spin labeling
α: labeling efficiency
BOLD: blood oxygen level dependent
B6C3: cross of C57BL/6 J and C3H/HeJ mice
CBF: cerebral blood flow
CVR: cerebrovascular reactivity
EPI: echo planar imaging
FA: flip angle
FLASH: fast low angle shot
FOV: field of view
fMRI: functional MRI
Iqr: interquartile range
λ: blood–brain partition coefficient of water
MRI: magnetic resonance imaging
M0b: magnetization of arterial blood
M0 t: magnetization of arterial tissue
pCASL: pseudo‐continuous arterial spin labeling
PLD: postlabel delay
RARE: rapid acquisition with relaxation enhancement
ROI: region of interest
SD: standard deviation
T: Tesla
τ: labeling duration
tc‐pCO_2_: partial pressure in transcutaneous pCO_2_
TE: echo time
TR: repetition time
TTHP: time to half peak
T1b: blood T1
T1 t: tissue T1

## 1 INTRODUCTION

Cerebral blood flow (CBF) and cerebrovascular reactivity (CVR) are emerging as potential biomarkers for cognitive impairment and dementia.^1^, ^2^ They can be measured in the clinic^3^ and in preclinical research^4^, ^5^ by means of MRI‐based perfusion imaging. The arterial spin labeling (ASL) MRI technique is particularly attractive as it is completely noninvasive, using blood water as an endogenous tracer. This enables repeated measures for dynamic recordings of responses to stimuli such as a “CO_2_ challenge” for assessment of CVR.

Mouse models are important tools for translational neuroscience and neurology. Although MRI studies with awake mice have been performed,^6^, ^7^ this approach is often tied to ethical and technical restrictions that heavily restrict practical application. Therefore, mouse MRI is commonly performed under anesthesia. However, anesthesia protocols are known to influence cerebral hemodynamics. In order to more clearly relate MRI measures of brain hemodynamics obtained from mouse models to clinical data, and for meaningful comparison with other preclinical studies, it is important to understand the hemodynamic influence of the different anesthesia protocols used. Furthermore, different mouse strains may vary in their response to the anesthesia protocol used.^8^, ^9^ It is therefore important to devise standardized protocols in order to provide comparable and reproducible CBF and CVR estimates. Isoflurane is the most widely used anesthetic in imaging studies with mice, probably due to its very straightforward method of administration, fast reversibility and the minimal long‐term side effects, even upon repeated use in the same animal.^10^ However, isoflurane also has a dose‐dependent vasodilatory effect,^11^ as well as a dose‐dependent respiratory depressive effect.^12^, ^13^ Thus, it has been posited to reduce the dynamic range in ASL‐based CVR studies, both directly via its vasodilatory effect and—if not ventilated—indirectly via an increase in the partial pressure of arterial pCO_2_ as a consequence of respiratory depression.

Blood oxygen level dependent functional MRI (BOLD‐fMRI) studies also appear to be impaired by isoflurane in a dose‐dependent manner, as stimulus‐evoked BOLD responses and resting state network organization have been shown to be reduced when higher levels of isoflurane were used.^14^, ^15^ In order to benefit from its otherwise favorable characteristics, refinements have been proposed to minimize the needed dose of isoflurane. For example, combining it with a low dose of medetomidine permits stable anesthesia at 0.5% of isoflurane.^16^ Also, isoflurane has been combined with the paralysis agent pancuronium bromide to support mechanical ventilation, with maintenance levels of 1.0%.^17^ However, combination with paralyzing agents or medetomidine may not always be wanted, due to ethical reasons or unresponsiveness of the mouse strain used, respectively.^9^ Alternatively, Wells et al showed that by simply administrating a relatively low concentration of isoflurane both during induction (2.0%) and maintenance (1.5%)—without any other anesthetic—workable CVR values can be obtained in an ASL‐based CVR study.^18^

In this work we provide the first comprehensive assessment of CBF and CVR in the mouse brain under different anesthetic regimes using pseudo‐continuous ASL‐MRI in C57BL/6 J mice. In particular, given the prevalence and unique perks of isoflurane anesthesia, we compare three distinct isoflurane‐based protocols: (i) a free‐breathing isoflurane protocol with doses that reflect commonly used isoflurane concentrations in mouse brain MRI studies,^9^, ^19^ defined henceforth as the “medium‐dose” protocol; (ii) the protocol described by Wells et al,^18^ where a relatively low dose of isoflurane was used for both induction and maintenance, defined henceforth as the “low‐dose” protocol; and (iii) a medium‐dose isoflurane protocol with mechanical ventilation to counteract the respiratory depressive effect. Additionally, we compare the three isoflurane protocols with a medetomidine anesthesia protocol, which is known to be vasoconstrictive. Furthermore, we evaluate the low‐dose and ventilated isoflurane protocols in a second mouse strain, partly on a C3H/HeJ background, a strain with known respiratory anomalies.^20^

## 2 EXPERIMENTAL

This study was performed in compliance with the guidelines of the European community for the care and use of laboratory animals (EUVD 86/609/EEC) and was reported conforming to the ARRIVE guidelines.^21^

### 2.1 Animal procedures

All experiments were approved by the local ethics committee “Instantie voor Dierenwelzijn” of the Leiden University Medical Center and were performed under DEC permits 11165 and 14073.

Thirty‐three wild‐type C57BL/6 J mice (31 males, two females) were used at 10.7 months (standard deviation [SD] 6.5 months), with a mean weight of 35 g (SD 6 g). The second wild‐type strain used in this study was the F2 generation of a cross of C57BL/6 J and C3H/HeJ mice, referred to henceforth as B6C3. Eleven B6C3 mice (seven males, four females) were used at 13.5 months (SD 0.5 months), with a mean weight of 46 g (SD 7 g). The mice were derived from an in‐house breeding. Founder mice were obtained from the Jackson Laboratory (Bar Harbor, ME, USA). The mice were co‐housed in individually ventilated cages (2–4 per cage) in a 12 h dark/light cycle‐ML2‐facility. They had unlimited chow food and water at their disposal and were supplied with bedding material and cage enrichment.

### 2.2 Anesthesia and monitoring of the physiological signals

The following four anesthesia protocols were used (dose, route of administration, animal number and age per group are summarized in Table S1):

1. An isoflurane protocol with commonly used doses in mouse brain MRI studies^9^, ^19^: 3.5% induction for four minutes and 1.5–2.0% maintenance; during maintenance the breathing rate was kept between 80 and 100 bpm by adjusting the isoflurane accordingly between 1.5–2.0%. The isoflurane was administered in medical air enriched with oxygen (air:oxygen 3:1). This protocol is referred to as the medium‐dose isoflurane protocol.
2. An isoflurane protocol with 2.0% induction for five minutes and 1.25% maintenance, based on the protocol described in Wells et al.^18^ However, to restrict the accumulation of isoflurane even more, the maintenance concentration was lowered to 1.25% (instead of 1.50% in Wells et al), which was the minimum in our study. Some B6C3 animals required slightly longer induction times to be sufficiently anesthetized. For both strains, a quick transfer from the induction box to the animal bed of the MRI set‐up was necessary to prevent awakening. The isoflurane was administered in pure medical air, in accordance with Wells et al.^18^ This protocol is referred to as the low‐dose isoflurane protocol.
3. An isoflurane protocol with 3.5% induction for four minutes, 1.75% maintenance and intubation and mechanical ventilation. After induction, the mouse was taken out of the induction box and transferred to a supine position and supplied with a nose cone with continued 3.5% isoflurane administration. The trachea was subsequently endotracheally intubated, after which the mouse was transferred to the scanner bed and the isoflurane was then decreased to 1.75%. There, the mechanical ventilation was started with a CWE MRI‐1 ventilator (Ardmore, OK, USA) with settings as recommended during the Functional MRI in Mice workshop in November 2016 at the animal imaging center of the ETH Zürich, Switzerland: a rate of 80 bpm, a tidal volume of 1.7 mL, and inspiration of 25%. The animal was not paralyzed during the scan. Both the gas used during induction and the air used during ventilation consisted of oxygen‐enriched medical air (air:oxygen 3:1). This protocol is referred to as the medium‐dose isoflurane + ventilation protocol.
4. A medetomidine protocol. Here, anaesthesia was also induced with 3.5% isoflurane in oxygen‐enriched medical air (air:oxygen 3:1) for four minutes. After transfer to the animal bed, the isoflurane was decreased to 2.0%, and after finalizing the set‐up, a subcutaneous catheter was inserted into the flank of the animal. Then a 0.15 mg/kg bolus of dexmedetomidine hydrochloride was given (Dexdomitor, Vetoquinol SA, Lure, France [a solution without levomedetomidine]), followed 10 minutes later by 0.30 mg/kg/h infusion with a syringe pump (Univentor 802, Univentor High Precision Instruments, Zejtun, Malta). Note that this is equivalent to a 0.30 mg/kg bolus and 0.60 mg/kg/h infusion protocol when a mixture of both active (dexmedetomidine) and inactive (levomedetomidine) enantiomers is used. During the 10 minutes between bolus and infusion, the isoflurane was decreased to 0%, but the administration of oxygen‐enriched air was continued. The protocol is based on the optimal findings in the medetomidine optimization study by Adamczak et al.^22^ This protocol is referred to as the medetomidine protocol.

For physiological monitoring, heart and respiration rates were captured with a pressure‐sensitive pad below the animal and a pulse oxygenation probe around the hind paw, respectively (SA Instruments, New York, NY, USA). Temperature was maintained around 36.5°C with a water bed plugged into a feedback control system (Medres, Cologne, Germany). Furthermore, a transcutaneous probe (Radiometer, Zoetermeer, the Netherlands) was applied on the shaved flank of the mouse for noninvasive collection of the transcutaneous partial pressure in carbon dioxide (tc‐pCO_2_). As absolute arterial pCO_2_ values are not reflected by the tc‐pCO_2_ values, whereas changes in arterial pCO_2_ are reflected by changes in tc‐pCO_2_,^23^ the tc‐pCO_2_ plots are shown as absolute change of tc‐pCO_2_ in mmHg during challenge relative to baseline. Due to a technical error during the medetomidine scans, seven out of the 11 tc‐pCO_2_ profiles were not captured in this group. For each anesthesia protocol, representative CBF, heart rate, respiration rate and tc‐pCO_2_ time‐profiles are shown in Figure S1. In a few animals, parts of the heart or respiration rate time‐profiles were not captured properly, as illustrated in Figure S1. Such incorrectly sampled data were excluded from the median and interquartile range (iqr) calculation of the heart and respiration rates, which is shown in Table S2.

### 2.3 MRI measurements

A 7 T Pharmascan (Bruker Biospin GmbH, Ettlingen, Germany) was used with a 23 mm volume resonator.

#### 2.3.1 Anatomical scans

For planning of the pCASL sequence, anatomical T2‐weighted magnitude images were acquired with a RARE sequence in the axial, sagittal and coronal orientation, using the following parameters: TE/TR/flip angle (FA) = 35.0 ms/2500 ms/90 degrees, matrix 256 x 256, FOV 21.55 x 21.55 mm, 0.7 mm slice thickness, no slice gap, 1 average, RARE factor of 8, and a bandwidth of 36.7 kHz. Additionally, a set of anatomical images with the same geometry as the pCASL read‐out was acquired for registration purposes.

#### 2.3.2 Cerebral blood flow measurements

A pCASL sequence enabled measurement of the cerebral blood flow. The pCASL‐interpulse phases were optimized to correct for off‐resonance effects with prescans.^24^ Labeling pulses were applied with 3.5 μT B1, 400/800 μs pulse duration/interval, and a total labeling duration (τ) of 3000 ms. A postlabel delay (PLD) of 300 ms was included before a single‐shot spin echo–echo planar imaging (EPI) read‐out. The following read‐out parameters were used: TE/FA = 16.8 ms/90 degrees, four dummy scans, matrix size 96 x 96, FOV 21.55 x 21.55 x 1.5 mm, three slices with the middle slice position at −0.75 mm Bregma and at the isocenter of the bore and a maximum/average labeling gradient of 45.0/5.0 mT/m. The labeling slice was always positioned exactly 1.0 cm from the middle imaging slice, which is around 4 mm upstream of the split of the common carotid artery. The TR was 3520 ms, meaning that each control/labeled pair of images took 7.04 seconds to acquire. An example of the planning and the resulting ASL images is shown in Figure S2.

To support CBF‐quantification, tissue T1 (T1 t) maps were acquired with an inversion recovery EPI sequence with 18 inversion times, and labeling efficiency (α) was measured in the carotids 3 mm downstream of the labeling plane with a flow‐compensated, ASL‐encoded FLASH sequence.

#### 2.3.3 Timeline of the CBF measurements

CBF time‐profiles were acquired for 21 minutes separated in three fragments of seven minutes: seven minutes baseline, seven minutes 7.5% CO_2_ challenge, seven minutes back to baseline. This duration was chosen to collect 60 repetitions for each fragment of the sequence, thus with a total of 180 repetitions. During the challenge, the oxygen concentration stayed the same, ie 7.5% CO_2_ replaced N_2_ in the air mixture.

### 2.4 Image processing

#### 2.4.1 Alignment of the pCASL and inversion recovery EPIs

A MATLAB monomodal rigid‐body registration (300 iterations) was applied on the label and control magnitude images collected by the pCASL sequence to align them to the first label magnitude image of the sequence. In the same way, the 18 inversion recovery EPIs were aligned to the first label magnitude image of the pCASL sequence. The CBF and T1 t maps could then be computed voxel‐wise.

#### 2.4.2 Cerebral blood flow quantification

The CBF was quantified in mL/100 g/min using Buxton's general kinetic perfusion model.^25^ Assuming that M0b, the magnetization of arterial blood at thermal equilibrium, may be approximated by M0 t/λ, where M0 t is the magnetization of tissue and λ is the blood–brain partition coefficient of water, ie 0.9 mL/g,^26^ the following equation was used:

(1) $$\mathrm{CBF}=\frac{\lambda \cdot \mathrm{\Delta M}\cdot \exp\left(\mathrm{PLD}/T_{1b}\right)}{2\cdot \alpha \cdot T_{1t}\cdot M_{0t}\cdot \left(1-\exp\left(-\tau /T_{1t}\right)\right)}$$

where ΔM is the measured difference between label and control acquisition and T1b is 2230 ms, the longitudinal relaxation time of blood at 7 T.^27^

#### 2.4.3 Delineation of the brain regions

The anatomical T2‐weighted magnitude images in one dataset of the study served as the basis for the delineation of the full brain and the cortex. These brain regions were then automatically propagated to the anatomical T2‐weighted magnitude images of the other datasets by applying a monomodal optical‐flow registration algorithm. The field of transport $\vec{V}$ was thus estimated on a pixel‐by‐pixel basis by means of the following minimization process:

(2) $$\arg\min_{\vec{V}}\int_{\Omega }\left|I_{t}+\vec{V}\cdot \vec{\nabla }I\right|+\alpha \left({\left\|\vec{\nabla }u\right\|}_{2}^{2}+{\left\|\vec{\nabla }v\right\|}_{2}^{2}\right)d\vec{r}$$

where *Ω* ⊆ *ℝ*^2^ is the image coordinate domain, *I*_*t*_ the temporal partial derivative of the image intensity *I* calculated between the time points *t* and *t* + δ*t*, (*u,v*) the estimated components of transport vectors, and $\vec{r}\;$∈ Ω the spatial location. The minimized functional accounts for the two following additive contributions: (i) a data fidelity term (left part of the integral in Equation (2)) that measures, through a *L* ^*1*^ *L* ^*1*^ norm, the similarity between the images, and (ii) a regularization term (right part of the integral in Equation (2)) designed to provide a sufficient conditioning to the numerical scheme. The regularization term is given by ${\left\|\vec{\nabla }u\right\|}_{2}^{2}=u_{x}^{2}+u_{y}^{2}$ and ${\left\|\vec{\nabla }v\right\|}_{2}^{2}=v_{x}^{2}+v_{y}^{2}$, *u*_*x*_ *u*_*x*_, *u* *u*_*y*__*y*_, *v*_*x*_ *v*_*x*_ and *v* *v*_*x*__*y*_ being the spatial partial derivatives of *u* and *v*, respectively. The regularization term alpha was set to 0.5. The results of this propagation step were then verified dataset‐by‐dataset by an operator (L.P. Munting).

In each dataset, the in‐plane spatial transformation between the anatomical T2‐weighted magnitude image and the first frame of the pCASL magnitude image (ie the reference image chosen previously) was then automatically estimated as follows: an edge‐based variational method for nonrigid multimodal registration^28^ was employed to circumvent potential geometrical distortions induced by the EPI readout. For this purpose, the data fidelity term in Equation (2) (left part of the integral) was replaced by a multimodal similarity metric which favors the alignment of edges/gradients that are present in both images, as described in Chen et al.^29^ The obtained motion‐vector field was subsequently employed to adjust the position of the masks of each brain region. Here again the results of this propagation step were verified dataset‐by‐dataset by an operator (L.P. Munting).

#### 2.4.4 CVR quantification

The spatial mean of the CBF in each brain region was collected frame‐by‐frame, which enabled quantification of the CVR by computing the following estimates: (i) baseline CBF from the mean CBF of the 20 repetitions, ie two minutes 20 seconds, before the onset of the CO_2_ challenge, and (ii) CBF during CO_2_ challenge based on the 20 repetitions before the end of the CO_2_ challenge. The percentage of CVR was calculated by applying the following equation:

(3) $$\mathrm{CVR}\;\left(\%\right)=100*\left(\;\frac{\text{mean}\;\mathrm{CBF}\;\text{during challenge}}{\text{mean baseline}\;\mathrm{CBF}}-1\right)$$

### 2.5 Statistical analysis

Estimates are expressed as median (iqr), except for the time‐profiles in the figures, which are given in mean (standard deviation [SD]) to better visualize the group response. The statistical tests were performed using IBM SPSS statistics software package version 23 (Armonk, New York, NY, USA). For multiple comparisons, initially a Kruskal‐Wallis test was used to evaluate whether the nonparametric unpaired groups belonged to the same distribution. If a significant difference was found, post hoc testing was performed with Dunn‐Bonferroni tests to assess which groups differed. Mann–Whitney tests were only used when comparing two groups.

## 3 RESULTS

### 3.1 Comparison of the anesthesia protocols in C57BL/6 J mice

**Figure 1:**
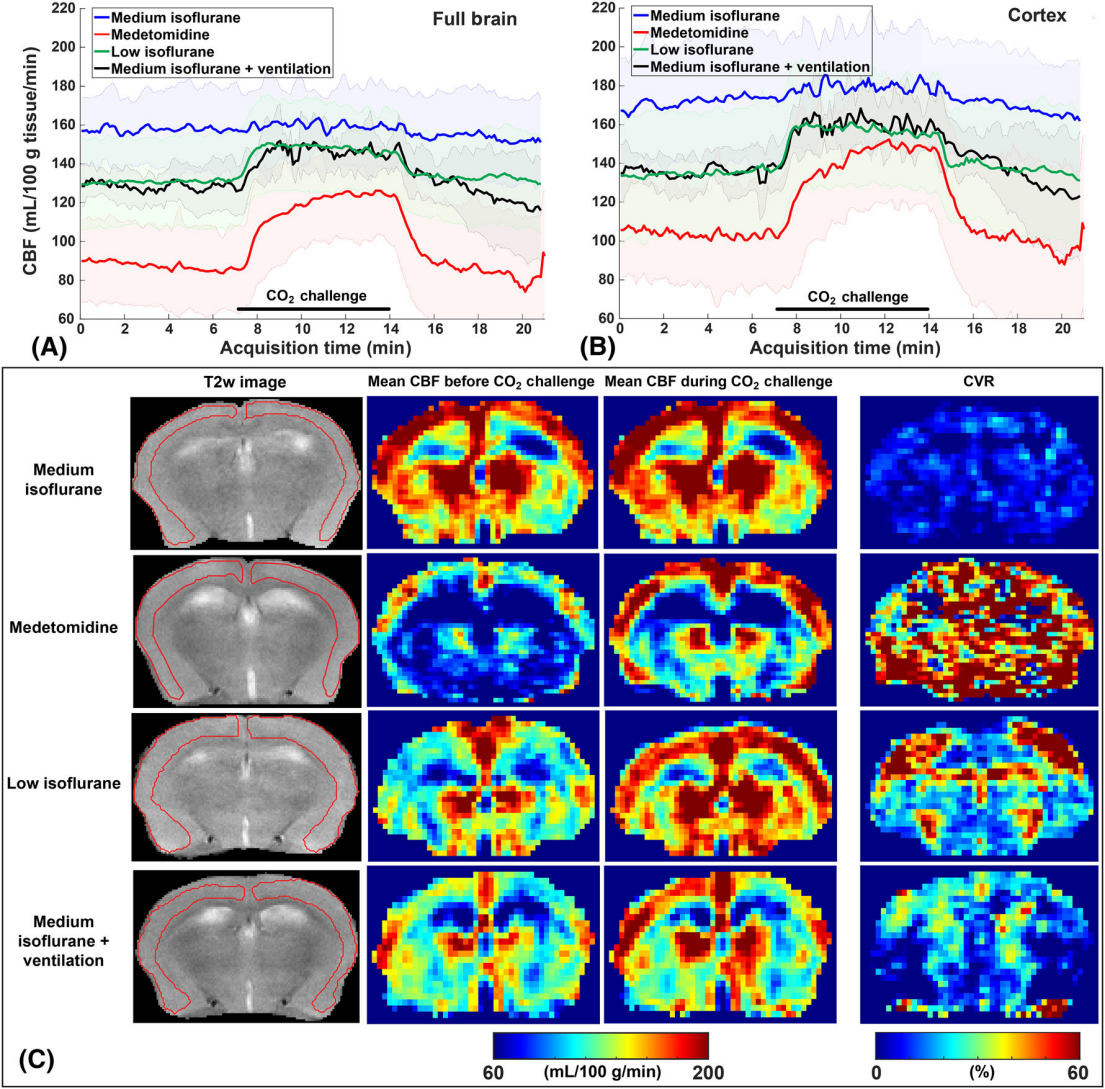
Absolute CBF values and CBF maps for the four different anesthesia protocols in C57BL/6 J mice. Mean and standard deviation of the absolute CBF profiles in (A) the full brain and in (B) the cortex. (C) Representative examples of images acquired at −0.75 mm from Bregma. In the columns from left to right: Anatomical images with cortical ROIs; mean CBF maps collected in the last two minutes before the onset of the CO_2_ challenge; mean CBF maps collected in the last two minutes during CO_2_ challenge; and CVR maps. All the images on a row are from the same mouse

**Figure 2:**
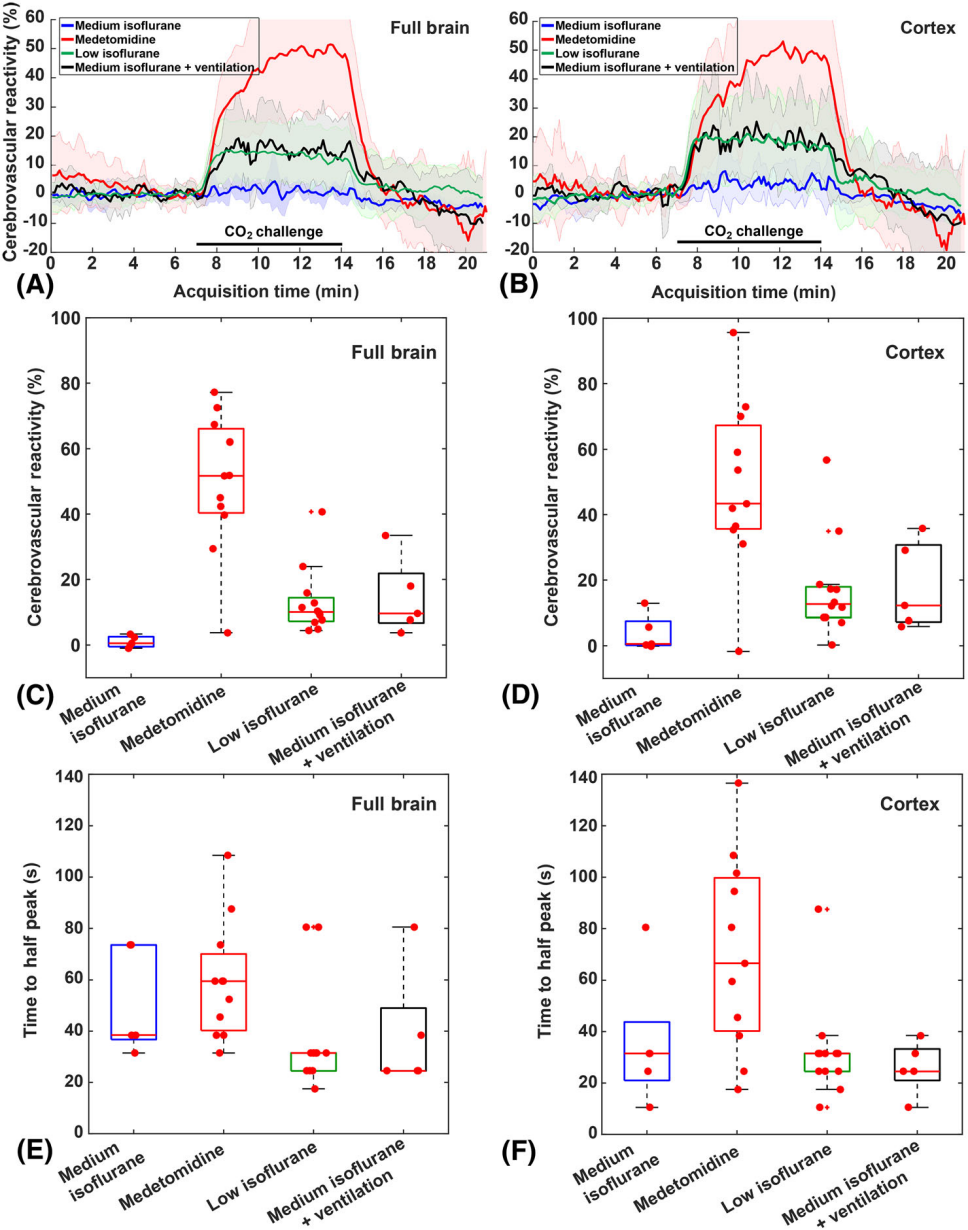
CVR relative to the baseline CBF for the four different anesthesia protocols in C57BL/6 J mice. CVR estimates are computed in the full brain (A, C, E) and in the cortex (B, D, F)

The CBF time‐profiles for the different anesthesia protocols are displayed in Figure 1A,B for full‐brain ROIs and cortical ROIs, respectively. CBF and CVR maps of individual mice representative of their groups and their respective anatomical T2‐weighted images are shown in Figure 1C. The lowest baseline CBF estimates were found using the medetomidine protocol, and the highest when administering medium isoflurane. The other two isoflurane protocols are in‐between, without an obvious difference between the two. Figure 2A,B shows CBF time‐profiles normalized to the baseline CBF values for the full brain and cortex, respectively. The resulting CVR values are compared in Figure 2C,D with boxplots for full brain and cortex, respectively. Here, an opposite trend is observed for the anesthesia protocols, with medium isoflurane resulting in the lowest CVR, and medetomidine resulting in the highest CVR. Again, the other two isoflurane protocols result in an intermediary CVR response, with similar results recorded.

The distribution of CBF and CVR values in both full brain and cortex for the four anesthesia protocols were significantly different from each other (H (3) = 19.02 and *P* < 0.001; H (3) = 24.24 and *P* < 0.001; H (3) = 12.36 and *P* = 0.006; H (3) = 14.75 and *P* = 0.002 for full‐brain CBF, full‐brain CVR, cortical CBF and cortical CVR, respectively). Post hoc analysis indicated that the CBF values in the full brain obtained under medetomidine (84.4 mL/100 g/min [31.4 mL/100 g/min]) were significantly lower than those obtained with medium isoflurane (157.7 mL/100 g/min [41.72 mL/100 g/min]) and significantly lower than those obtained under low isoflurane (138.9 mL/100 g/min [26.5 mL/100 g/min]), adjusted *P* = 0.001 and adjusted *P* = 0.006, respectively. Regarding the CVR values in the full brain, the same groups differed: medium isoflurane (0.5% [3.1%]) vs medetomidine (51.7% [25.7%]), adjusted *P* < 0.001; low isoflurane (10% [7.1%]) vs medetomidine, adjusted *P* = 0.036. Most of the other comparisons lost their statistical significance after Dunn‐Bonferoni correction. For the low isoflurane and medium isoflurane + ventilation CBF and CVR comparisons, however, even uncorrected *P*‐values were not significant (0.688 and 0.974, respectively), reflecting their similar CBF time‐profiles. In the cortical ROIs, statistical testing gave comparable results as in the full‐brain ROIs (data not shown). Median and iqrs of the CBF and CVR values per anesthesia protocol are given in Table S3.

It should be pointed out that in the medetomidine group, not all of the individual baseline CBF profiles were stable yet, even though the medetomidine infusion started 20 minutes before the pCASL scan (see Figure S4). It is also to be noted that, unlike the isoflurane anesthesia protocols where the CVR is most pronounced in the cortex, the CVR obtained using the medetomidine protocol was strongly present throughout the full brain. Also observed in the CBF time‐profile of medetomidine only is a continuing CBF increase during hypercapnia, suggesting a competition between processes (vasoconstrictive medetomidine and vasodilative CO_2_) influencing the CBF. This is reflected in the time to half peak (TTHP) distributions, which were also different between the anesthesia protocols, H (3) = 9.701 and *P* = 0.021 in the full brain and H (3) = 9.105 and *P* = 0.028 in the cortex. Only the medetomidine vs low isoflurane full‐brain TTHP post hoc test survived the Dunn‐Bonferoni correction, with an adjusted *P* = 0.03.

There were no significant differences in the tc‐pCO_2_ rise upon CO_2_ challenge between the different anesthesia protocols (Figure S3, H (3) = 6.167; *P* = 0.104). The median heart rates were lower and respiration rates higher in the medetomidine group (Table S2), which was to be expected given the known respiratory depressive action of isoflurane^12^, ^13^ and the known bradycardic action of medetomidine.^30^

### 3.2 Comparison of the anesthesia protocols in B6C3 vs C57BL/6 J mice

**Figure 3:**
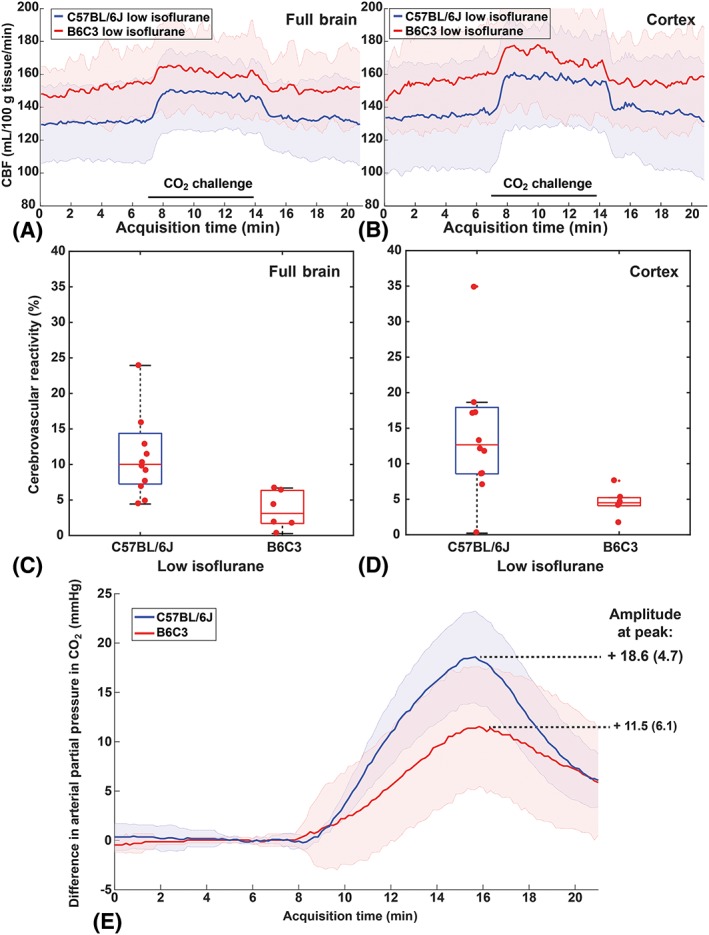
CBF and CVR values for the “low isoflurane” protocol in C57BL/6 J mice (blue) vs B6C3 mice (red). Time‐profiles of the CBF acquired in (A) the full brain and in (B) the cortex. (C, D) the CVR is significantly different between both strains. (E) the transcutaneous pCO_2_ rise was significantly different at the time of maximum pCO_2_ rise

The B6C3 mice could not be sedated sufficiently with medetomidine, thus compromising the use of this anesthetic for this strain. The application of the low isoflurane protocol in free‐breathing B6C3 mice showed a higher but nonsignificant mean baseline CBF in B6C3 mice compared with C57BL/6 J mice (Figure 3; full brain: U = 21.0, Z = −1.405, *P* = 0.180; cortex: U = 24.0, Z = −1.124, *P* = 0.291). Using the low isoflurane protocol, a significantly lower CVR in B6C3 mice was found vs C57BL/6 J mice: 3.1% (4.6%) vs 10.0% (7.1%) in the full brain (U = 4.0, Z = −2.997, *P* = 0.001); and 4.5% (1.1%) vs 12.7% (9.3%) in the cortex (U = 7.0, Z = −2.716, *P* = 0.005) (Figure 3A‐D). Interestingly, the change in tc‐pCO_2_ was also significantly lower in B6C3: mean rise of 18.6 mmHg in C57BL/6 J mice vs 11.5 mmHg in B6C3 mice (Mann–Whitney, *P* = 0.014) (Figure 3E). It should be noted that B6C3 mice displayed a higher group variability in tc‐pCO_2_ rise, with a SD of 6.1 mmHg (normalized SD of 53%) vs 4.7 mmHg only in C57BL/6 mice (normalized SD of 25%) (Figure 3E).

**Figure 4:**
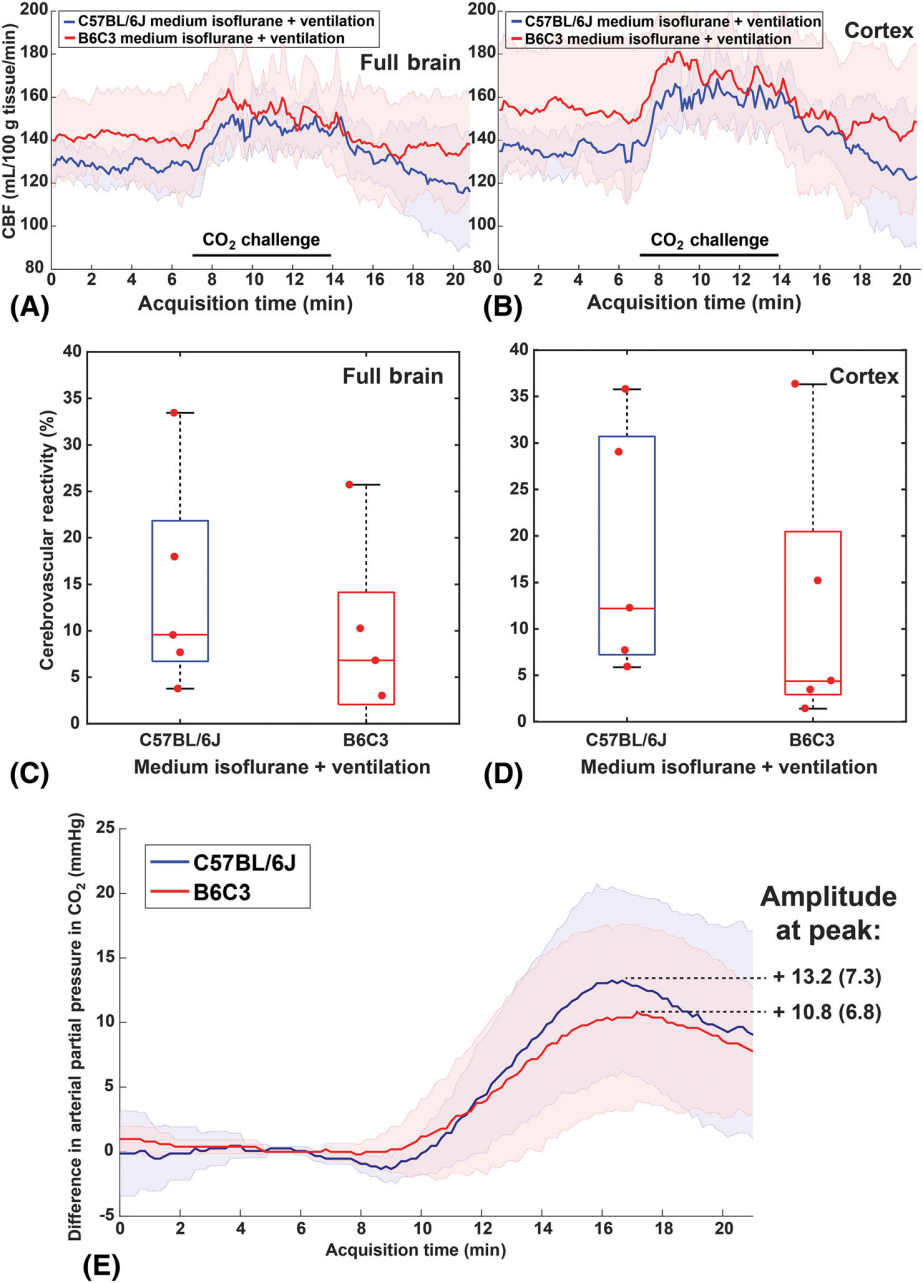
CBF and CVR for the “standard isoflurane + mechanical ventilation” protocol: C57BL/6 J mice (blue) vs B6C3 mice (red). Time‐profiles of the CBF acquired in (A) the full brain and in (B) the cortex. CVR in (C) the full brain and in (D) the cortex, which are not significantly different in B6C3 vs C57BL/6 J mice. (E) Tc‐pCO_2_ rise in B6C3 vs C57BL/6 J mice was not significantly different

When using medium isoflurane + mechanical ventilation, the difference in CVR in B6C3 mice vs C57BL/6 J mice was abolished: 6.8% (12.1%) vs 9.6% (15.1%) in the full brain (U = 8.0, Z = −0.940, *P* = 0.421), and 4.4% (17.5%) vs 12.2% (23.5%) in the cortex (U = 8.0, Z = −0.940, *P* = 0.421) (Figure 4A‐D). With mechanical ventilation, both strains displayed a similar tc‐pCO_2_ rise, with a mean rise of 13.2 mmHg (7.3) vs 10.8 mmHg (6.8) in B6C3 mice (t‐test, *P* = 0.595) (Figure 4E).

### 3.3 End of the hemodynamic capacity reserve with high baseline CBF values

**Figure 5:**
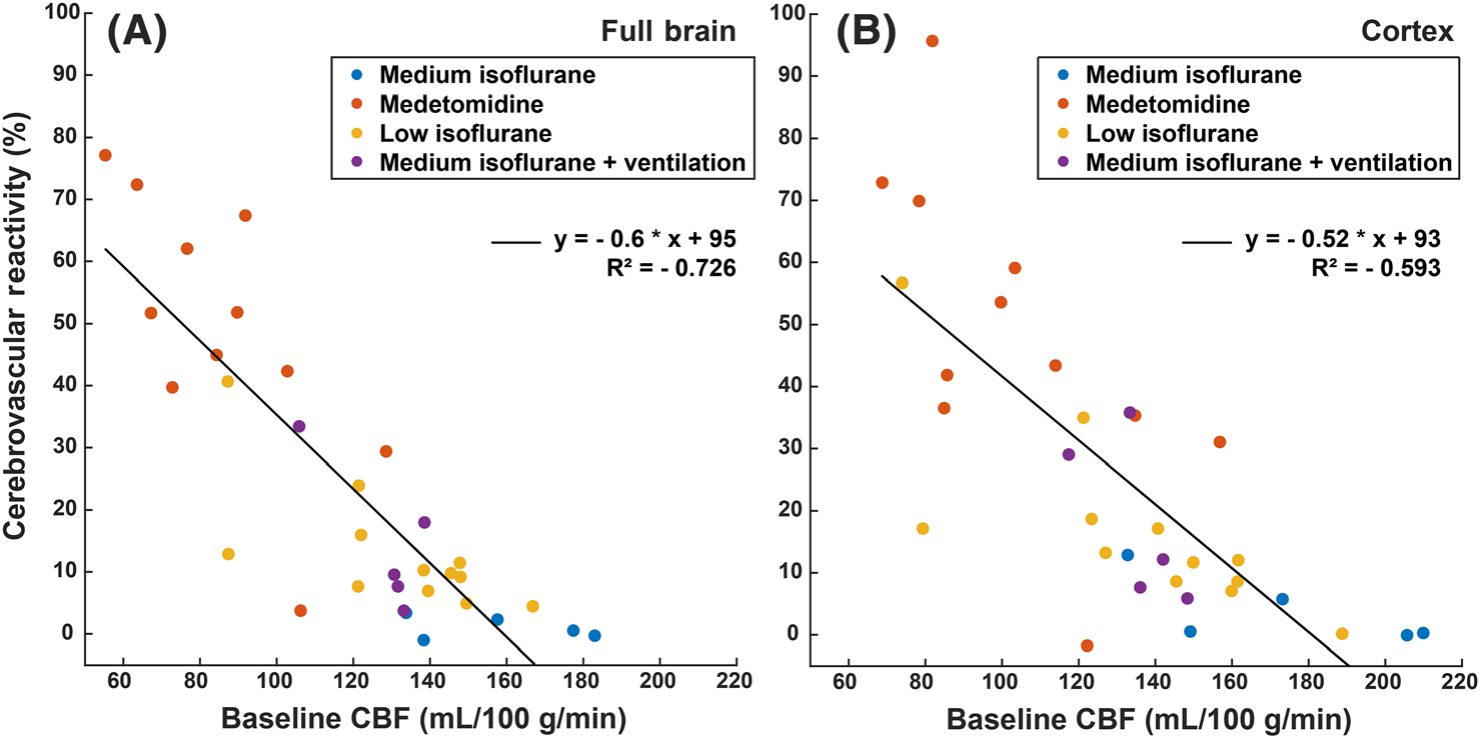
CVR expressed as a function of the baseline CBF in C57BL/6 J mice. Each dot represents one mouse, where the colour indicates the anesthesia protocol used. Relations are given for (A) the full brain and (B) the cortex

To visualize the dependency of CVR on baseline CBF, a correlation plot between the two is shown in Figure 5. There is a strong negative correlation between baseline CBF and CVR, with high baseline CBF values resulting in a blunted or absent CVR response regardless of the protocol used, indicating the maximum of the hemodynamic capacity is reached. Only measurements in C57BL/6 J mice were used for this plot.

## 4 DISCUSSION

The current increase of studies investigating hemodynamics in the mouse brain requires a better understanding of the physiological influence of the anesthesia protocols used. Our results demonstrate exquisite sensitivity of CBF and CVR to the anesthesia protocol employed, findings that reinforce the need for care in the experimental design and interpretation of studies of murine cerebrovascular function, where anesthesia is often required. Isoflurane is the most widely used anesthestic due to its many advantages, but it has the disadvantage of being a dose‐dependent vasodilator and a respiratory depressant. In this study, we focused on the influence of different isoflurane protocols on the hemodynamic measures CBF and CVR to a CO_2_ stimulus. A medium‐dose isoflurane protocol was compared with two alternative isoflurane protocols that have been adapted to minimize its vasodilatory and/or respiratory depressive properties: a low‐dose isoflurane protocol and a medium‐dose isoflurane protocol with mechanical ventilation. To put the results in context, the isoflurane protocols were also compared with the vasoconstrictive medetomidine protocol. All four protocols were tested in the commonly used C57BL/6 J mouse strain. Lastly, the performance of the adapted isoflurane protocols were also assessed in a second mouse strain, namely, the mixed background B6C3 strain.

As reported previously,^9^ the medium isoflurane protocol resulted in a high baseline CBF, thus blunting the CVR. Conversely, the medetomidine protocol yielded the lowest CBF and highest CVR, with median CVR estimates over 50% in C57BL/6 J mice. As intended, the adapted isoflurane protocols lowered basal CBF compared with the medium isoflurane protocol. Consequently, workable CVR values were obtained. There was no observable difference between the two adapted isoflurane protocols. This is interesting, because with the low isoflurane protocol, both the vasodilatory and the respiratory depressive effects are reduced compared with the medium isoflurane protocol, whereas with the medium isoflurane + ventilation protocol, only the respiratory depressive effects are reduced. Thus, it implies that the respiratory depression plays a major role in the outcome of the high baseline CBF with the medium isoflurane protocol. Because of its much easier implementation, we recommend researchers unexperienced with intubation the low isoflurane protocol over the medium isoflurane + ventilation protocol for CVR studies with perfusion imaging. It should be stressed here, that in our experience, not only the low maintenance, but also the low induction is of extreme importance for maintaining some hemodynamic reserve capacity. Compared with the other protocols, the low induction keeps the respiration rate higher, especially during the beginning of the anesthesia period, and fully avoids any spasmodic breathing. Also to be noted is that studies have been reported with lower maintenance concentrations than our low isoflurane protocol.^6^, ^14^ In our study we could not lower the isoflurane concentration, as the animals would then have woken up. Perhaps local differences in isoflurane delivery systems can explain differences in lower boundaries of isoflurane maintenance. In Table S4, a list of advantages and disadvantages of the different anesthesia protocols in this study are summarized.

A wider range of anesthetics has been used for rodent MRI studies than was used here. Most of these anesthetics have been reported to affect hemodynamic regulation. For example, propofol reduces blood pressure and is respiratory depressive.^31^, ^32^ Other fluorinated gases than isoflurane such as halothane and sevoflurane have a similar hemodynamic influence as isoflurane.^33^ Of particular interest are urethane and etomidate. These anesthetics are known to have very little effect on hemodynamics.^9^, ^34^, ^35^ However, urethane is not applicable to longitudinal studies, because of the toxicity of the compound. Etomidate sedation has been suggested as a widely applicable anaesthesia regime in multiple strains.^9^, ^35^ It is even preferred for anesthesia induction of cardiac patients, because of its lack of hemodynamic influence.^36^ However, the current availability of formulations of etomidate is very limited and, to the best of our knowledge, etomidate is only available in a concentration of 2 mg/mL. This means that injection volumes needed for at least one hour of sedation exceed the animal welfare guidelines.^37^ A recent paper showed that it was possible to measure unilateral BOLD responses upon a somatosensory stimulus repeatedly in the same mouse anesthesized with a novel ketamine/xylazine formulation.^38^ It would therefore be of interest to characterize the absolute CBF and CVR profiles with this new ketamine/xylazine formulation.

Unfortunately, B6C3 mice exhibited an insufficient anesthetic depth with medetomidine, thus compromising the use of this anesthetic for this strain. This insensitivity probably comes from the C3H strain. Medetomidine has been reported to show limited efficacy of anaesthesia, not only in C3H mice, but also in BTRB T + tf/J mice and CD1 strains.^9^ Therefore, if medetomidine use is uncharacterized in a mouse strain, we recommend careful bench studies with frequent monitoring of anesthetic depth and physiological measures such as heart and respiration rate before starting an MRI experiment with medetomidine. Another limitation of medetomidine is the unstable baseline CBF that was sometimes observed in the C57Bl/6 J mice. This is probably a result of the switch from isoflurane induction to medetomidine. The dynamics of CBF stabilization under medetomidine are not well known and this could be an interesting subject for future research. However, because the sedation time of medetomidine is limited (around one hour starting from the time of bolus injection),^22^ it would be risky to delay the start of the pCASL scan as the animal may wake up before the end of the scan.

A different complication with the CVR measurements was encountered when using the low isoflurane protocol in free‐breathing B6C3 mice. Here, a CBF response to CO_2_ was nearly absent (3.1% CVR). Interestingly, when comparing the mean tc‐pCO_2_ increase with the same hypercapnia protocol in free‐breathing mice, B6C3 mice also had a significantly lower tc‐pCO_2_ increase compared with C57BL/6 J mice (11.5 mmHg vs 18.6 mmHg). When the B6C3 mice were mechanically ventilated, the differences in both CVR and tc‐pCO_2_ between B6C3 and C57BL/6 J mice were abolished. It has been reported before that C3H mice have a low respiratory sensitivity to hypercapnic stimulation.^20^ However, it seems paradoxical that a lower tc‐pCO_2_ increase is measured in free‐breathing mice with a lower respiratory response to CO_2_, as a lower respiratory response is expected to less efficiently remove CO_2_ from the body. Further research is therefore necessary to clarify if and how the reduced CVR, reduced tc‐pCO_2_ rise, and low respiratory sensitivity to hypercapnia in the B6C3 mice anesthesized with low isoflurane are related. Whole‐body plethysmography to measure minute ventilation as well as more reliable pCO_2_ measurements such as end‐tidal CO_2_ or blood sampling could provide more insight to this matter. Also to be taken into account is that factors such as skin perfusion influence the diffusion of CO_2_ through the skin,^23^ which might in turn be influenced by the anesthesia protocol used. This may bias the tc‐pCO_2_ measurements, which is why we chose not to express CVR per mmHg rise of transcutaneous CO_2_.

A few limitations specific to this study should be taken into account. The large age range of the animals and the different oxygen content in the low isoflurane protocol (20% vs around 40% O_2_ in the other protocols) might have biased the results. However, this bias is probably small, as several studies have shown that brain hemodynamic parameters are preserved with age in wild‐type mice,^5^, ^39^, ^40^ and because the effect of 100% O_2_ inhalation on the baseline CBF has been shown to be an order of magnitude lower than that of hypercapnia.^41^ Another limitation is that, because of technical constraints, the labeling efficiency was only measured in the carotids, and not in the vertebral arteries. This might have introduced noise in the CBF values of the flow territory of the vertebral arteries (the posterior part of the brain), but this is not expected to be different between groups. Lastly, the absence of paralysis in the mechanically ventilated animals sometimes resulted in counter‐breathing, especially during the CO_2_ administration, probably explaining the greater fluctuations in CBF time‐profiles in this group.

In conclusion, this study stresses the importance of careful selection of the specific anesthesia protocol for CBF and/or CVR studies in the mouse brain. From the four protocols tested, the medetomidine protocol showed the lowest CBF and highest CVR values, as was expected given medetomidine's vasoconstrictive properties. However, an important limitation is that medetomidine induces insufficient depth of anaesthesia in several mouse strains. Despite the known vasodilative properties of isoflurane, CVR studies were possible with the two adapted isoflurane protocols, with comparable CVR values in C57BL/6 J mice. Of these two, the free‐breathing low isoflurane protocol may experimentally provide the most straightforward protocol. With this protocol, it is important that the isoflurane dose is kept low during both induction and maintenance. Furthermore, CVR was shown to be highly dependent on the baseline CBF, with high baseline CBF resulting in blunted CVR.

## Supporting information


**Table S1.** **Anesthesia protocols used in this study**. Animal numbers and age apply to C57BL/6 J only.
**Table S2**. Median and interquartile ranges (IQR) of respiration and heart rates for the different anesthesia protocols and mouse strains.
**Table S3**. Median and interquartile ranges (IQR) of Cerebral Blood Flow (CBF), Cerebrovascular Reactivity (CVR) and Time to Half Peak (TTHP) values for the different anesthesia protocols used. Values apply to C57Bl/6 J mice only.
**Table S4**. Pros and cons of the anesthesia protocols tested.
**Figure S1.** Examples of physiological time‐profiles. Note the incorrect sampling of the heart rate in the first half of the time‐profile of the low isoflurane protocol. Also note the doubling of the measured respiration rate (ventilated at 80 bpm) in the ventilation protocol, due to extra breaths/movements that the mouse exhibited in sync with the ventilation protocol.
**Figure S2.** Example of the ASL spatial planning and resulting ASL images. The planning is shown on sagittal (upper left) and coronal (upper right) T2‐weighted RARE images. The label slice is depicted in green, the inversion efficiency imaging slice in red and the imaging slices in blue. The yellow arrow indicates the location of one of the two carotid arteries. Shown below is one pair of ASL images (both label and control), corresponding to the middle slice shown on the planning. Also shown is the relative difference of the two (subtraction of the label from the control, divided by the control).
**Figure S3.** The transcutaneous pCO2 profiles measured during the pCASL scans in the C57BL/6 J mice. The profiles are grouped per anesthesia protocol (mean ± SD). No significant differences were found between the groups, H (3) = 6.167; p = 0.104. Note that it takes around 2 minutes before the arterial CO2 has diffused to the skin (CO2 was administered from minute 7–14). Also to be mentioned is that 7 out of the 11 medetomidine profiles have not been captured due to a technical error.
**Figure S4.** Individual time‐profiles of the animals in the medetomidine group. Every line represents the results of the full brain ROI from one mouse. The red line indicates the group average. Note that some of the individual profiles are not stable yet in the first minutes of the scan.

## References

[1] Beishon L , Haunton VJ , Panerai RB , et al. Cerebral hemodynamics in mild cognitive impairment: a systematic review. J Alzheimers Dis. 2017;59:369‐385. 28671118 10.3233/JAD-170181

[2] Hays CC , Zlatar ZZ , Wierenga CE . The utility of cerebral blood flow as a biomarker of preclinical Alzheimer's disease. Cell Mol Neurobiol. 2016;36:167‐179. 26898552 10.1007/s10571-015-0261-z PMC5278904

[3] Alsop DC , Detre JA , Golay X , et al. Recommended implementation of arterial spin‐labeled perfusion MRI for clinical applications: a consensus of the ISMRM perfusion study group and the European consortium for ASL in dementia. Magn Reson Med. 2015;73:102‐116. 24715426 10.1002/mrm.25197 PMC4190138

[4] Williams DS , Detre JA , Leigh JS , et al. Magnetic resonance imaging of perfusion using spin inversion of arterial water. Proc Natl Acad Sci U S A. 1992;89:212‐216. 1729691 10.1073/pnas.89.1.212 PMC48206

[5] Maier FC , Wehrl HF , Schmid AM , et al. Longitudinal PET‐MRI reveals β‐amyloid deposition and rCBF dynamics and connects vascular amyloidosis to quantitative loss of perfusion. Nat Med. 2014;20:1485‐1492. 25384087 10.1038/nm.3734

[6] Jonckers E , Delgado y Palacios R , Shah D , et al. Different anesthesia regimes modulate the functional connectivity outcome in mice. Magn Reson Med. 2014;72:1103‐1112. 24285608 10.1002/mrm.24990

[7] Yoshida K , Mimura Y , Ishihara R , et al. Physiological effects of a habituation procedure for functional MRI in awake mice using a cryogenic radiofrequency probe. J Neurosci Methods. 2016;274:38‐48. 27702586 10.1016/j.jneumeth.2016.09.013

[8] Zuurbier CJ , Emons VM , Ince C . Hemodynamics of anesthetized ventilated mouse models: aspects of anesthetics, fluid support, and strain. Am J Physiol Heart Circ Physiol. 2002;282:H2099‐H2105. 12003817 10.1152/ajpheart.01002.2001

[9] Petrinovic MM , Hankov G , Schroeter A , et al. A novel anesthesia regime enables neurofunctional studies and imaging genetics across mouse strains. Sci Rep. 2016;6:24523. 27080031 10.1038/srep24523 PMC4832200

[10] Hohlbaum K , Bert B , Dietze S , et al. Severity classification of repeated isoflurane anesthesia in C57BL/6JRj mice‐assessing the degree of distress. PLoS One. 2017;12:e0179588. 28617851 10.1371/journal.pone.0179588 PMC5472303

[11] Matta BF , Heath KJ , Tipping K , et al. Direct cerebral vasodilatory effects of sevoflurane and isoflurane. Anesthesiology. 1999;91:677‐680. 10485778 10.1097/00000542-199909000-00019

[12] Eger EI . Isoflurane: a review. Anesthesiology. 1981;55:559‐576. 7027831 10.1097/00000542-198111000-00014

[13] Cesarovic N , Nicholls F , Rettich A , et al. Isoflurane and sevoflurane provide equally effective anaesthesia in laboratory mice. Lab Anim. 2010;44:329‐336. 20507878 10.1258/la.2010.009085

[14] Nair G , Duong TQ . Echo‐planar BOLD fMRI of mice on a narrow‐bore 9.4 T magnet. Magn Reson Med. 2004;52:430‐434. 15282829 10.1002/mrm.20158 PMC2949950

[15] Bukhari Q , Schroeter A , Rudin M . Increasing isoflurane dose reduces homotopic correlation and functional segregation of brain networks in mice as revealed by resting‐state fMRI. Sci Rep. 2018;8:10591. 30002419 10.1038/s41598-018-28766-3 PMC6043584

[16] Grandjean J , Schroeter A , Batata I , et al. Optimization of anesthesia protocol for resting‐state fMRI in mice based on differential effects of anesthetics on functional connectivity patterns. Neuroimage. 2014;102:838‐847. 25175535 10.1016/j.neuroimage.2014.08.043

[17] Schroeter A , Schlegel F , Seuwen A , et al. Specificity of stimulus‐evoked fMRI responses in the mouse: the influence of systemic physiological changes associated with innocuous stimulation under four different anesthetics. Neuroimage. 2014;94:372‐384. 24495809 10.1016/j.neuroimage.2014.01.046

[18] Wells JA , Holmes HE , O'Callaghan JM , et al. Increased cerebral vascular reactivity in the tau expressing rTg4510 mouse: evidence against the role of tau pathology to impair vascular health in Alzheimer's disease. J Cereb Blood Flow Metab. 2015;35:359‐362. 25515210 10.1038/jcbfm.2014.224 PMC4348392

[19] Kober F , Duhamel G , Callot V . Cerebral perfusion MRI in mice. Methods Mol Biol. 2011;771:117‐138. 21874475 10.1007/978-1-61779-219-9_6

[20] Tankersley CG , Fitzgerald RS , Kleeberger SR . Differential control of ventilation among inbred strains of mice. Am J Physiol. 1994;267:R1371‐R1377. 7977867 10.1152/ajpregu.1994.267.5.R1371

[21] Kilkenny C , Browne WJ , Cuthill IC , et al. Improving bioscience research reporting: the ARRIVE guidelines for reporting animal research. PLoS Biol. 2010;8:e1000412. 20613859 10.1371/journal.pbio.1000412 PMC2893951

[22] Adamczak JM , Farr TD , Seehafer JU , et al. High field BOLD response to forepaw stimulation in the mouse. Neuroimage. 2010;51:704‐712. 20211267 10.1016/j.neuroimage.2010.02.083

[23] Stout RW , Cho DY , Gaunt SD , et al. Transcutaneous blood gas monitoring in the rat. Comp Med. 2001;51:524‐533. 11924815

[24] Hirschler L , Debacker CS , Voiron J , et al. Interpulse phase corrections for unbalanced pseudo‐continuous arterial spin labeling at high magnetic field. Magn Reson Med. 2018;79:1314‐1324. 28585234 10.1002/mrm.26767

[25] Buxton RB , Frank LR , Wong EC , et al. A general kinetic model for quantitative perfusion imaging with arterial spin labeling. Magn Reson Med. 1998;40:383‐396. 9727941 10.1002/mrm.1910400308

[26] Herscovitch P , Raichle ME . What is the correct value for the brain–blood partition coefficient for water? J Cereb Blood Flow Metab. 1985;5:65‐69. 3871783 10.1038/jcbfm.1985.9

[27] Dobre MC , Uğurbil K , Marjanska M . Determination of blood longitudinal relaxation time (T1) at high magnetic field strengths. Magn Reson Imaging. 2007;25:733‐735. 17540286 10.1016/j.mri.2006.10.020

[28] Denis de Senneville B , Zachiu C , Ries M , et al. EVolution: an edge‐based variational method for non‐rigid multi‐modal image registration. Phys Med Biol. 2016;61:7377‐7396. 27694705 10.1088/0031-9155/61/20/7377

[29] Chen X , Gilkeson RC , Fei B . Automatic 3D‐to‐2D registration for CT and dual‐energy digital radiography for calcification detection. Med Phys. 2007;34:4934‐4943. 18196818 10.1118/1.2805994 PMC2743028

[30] Pan W , Hua X , Wang Y , et al. Dose response of dexmedetomidine‐induced resistance to hypoxia in mice. Mol Med Rep. 2016;14:3237‐3242. 27498747 10.3892/mmr.2016.5588

[31] Zuurbier CJ , Koeman A , Houten SM , et al. Optimizing anesthetic regimen for surgery in mice through minimization of hemodynamic, metabolic, and inflammatory perturbations. Exp Biol Med (Maywood). 2014;239:737‐746. 24668552 10.1177/1535370214524877

[32] Zeller A , Arras M , Lazaris A , et al. Distinct molecular targets for the central respiratory and cardiac actions of the general anesthetics etomidate and propofol. FASEB j. 2005;19:1677‐1679. 16046472 10.1096/fj.04-3443fje

[33] Crawford MW , Lerman J , Saldivia V , et al. Hemodynamic and organ blood flow responses to halothane and sevoflurane anesthesia during spontaneous ventilation. Anesth Analg. 1992;75:1000‐1006. 1443679 10.1213/00000539-199212000-00021

[34] Maggi CA , Meli A . Suitability of urethane anesthesia for physiopharmacological investigations in various systems. Part 2: cardiovascular system. Experientia. 1986;42:292‐297. 3007197 10.1007/BF01942510

[35] Wang Z , Schuler B , Vogel O , et al. What is the optimal anesthetic protocol for measurements of cerebral autoregulation in spontaneously breathing mice? Exp Brain Res. 2010;207:249‐258. 20972776 10.1007/s00221-010-2447-4

[36] Gooding JM , Weng JT , Smith RA , et al. Cardiovascular and pulmonary responses following etomidate induction of anesthesia in patients with demonstrated cardiac disease. Anesth Analg. 1979;58:40‐41. 571221 10.1213/00000539-197901000-00016

[37] Morton DB , Jennings M , Buckwell A , et al. Refining procedures for the administration of substances. Lab Anim. 2001;35:1‐41. 10.1258/0023677011911345 11201285

[38] Shim H‐J , Jung WB , Schlegel F , et al. Mouse fMRI under ketamine and xylazine anesthesia: robust contralateral somatosensory cortex activation in response to forepaw stimulation. Neuroimage. 2018;177:30‐44. 29730495 10.1016/j.neuroimage.2018.04.062

[39] Hirschler L , Munting LP , Khmelinskii A , et al. Transit time mapping in the mouse brain using time‐encoded pCASL. NMR Biomed. 2018;31:e3855. 10.1002/nbm.3855 29160952

[40] Tong X‐K , Lecrux C , Hamel E , et al. Age‐dependent rescue by simvastatin of Alzheimer's disease cerebrovascular and memory deficits. J Neurosci. 2012;32:4705‐4715. 22492027 10.1523/JNEUROSCI.0169-12.2012 PMC6620905

[41] Matsuura T , Fujita H , Kashikura K , et al. Modulation of evoked cerebral blood flow under excessive blood supply and hyperoxic conditions. Jpn J Physiol. 2000;50:115‐123. 10866703 10.2170/jjphysiol.50.115

