# Supplementary material for: Influence of different isoflurane anesthesia protocols on murine cerebral hemodynamics measured with pseudo‐continuous arterial spin labeling
Source: NMR Biomed. 2019 Jun 7;32(8):e4105. doi: 10.1002/nbm.4105 (PMC6772066; doi:10.1002/nbm.4105)
Supplement: Supplementary file 1 — Table S1. Anesthesia protocols used in this study. Animal numbers and age apply to C57BL/6 J only. Table S2. Median and interquartile ranges (IQR) of respiration and heart rates for the different anesthesia protocols and mouse strains. Table S3. Median and interquartile ranges (IQR) of Cerebral Blood Flow (CBF), Cerebrovascular Reactivity (CVR) and Time to Half Peak (TTHP) values for the different anesthesia protocols used. Values apply to C57Bl/6 J mice only. Table S4. Pros and cons of the anesthesia protocols tested. Figure S1. Examples of physiological time‐profiles. Note the incorrect sampling of the heart rate in the first half of the time‐profile of the low isoflurane protocol. Also note the doubling of the measured respiration rate (ventilated at 80 bpm) in the ventilation protocol, due to extra breaths/movements that the mouse exhibited in sync with the ventilation protocol. Figure S2. Example of the ASL spatial planning and resulting ASL images. The planning is shown on sagittal (upper left) and coronal (upper right) T2‐weighted RARE images. The label slice is depicted in green, the inversion efficiency imaging slice in red and the imaging slices in blue. The yellow arrow indicates the location of one of the two carotid arteries. Shown below is one pair of ASL images (both label and control), corresponding to the middle slice shown on the planning. Also shown is the relative difference of the two (subtraction of the label from the control, divided by the control). Figure S3. The transcutaneous pCO2 profiles measured during the pCASL scans in the C57BL/6 J mice. The profiles are grouped per anesthesia protocol (mean ± SD). No significant differences were found between the groups, H (3) = 6.167; p = 0.104. Note that it takes around 2 minutes before the arterial CO2 has diffused to the skin (CO2 was administered from minute 7–14). Also to be mentioned is that 7 out of the 11 medetomidine profiles have not been captured due to a technical error. Figure S4. Indi [file NBM-32-na-s001.docx]

**Supplementary tables**

**Supplementary table 1.** **Anesthesia protocols used in this study**. Animal numbers and age apply to C57BL/6J only.

| Anesthesia | Isoflurane induction | Maintenance | Mixture air:oxygen | Number of animals | Age in months - mean (stdev) |
| --- | --- | --- | --- | --- | --- |
| Standard isoflurane | 3.5 % | 1.5 – 2.0 % | 3:1 | 5 | 8.0 (2.8) |
| Low-dose isoflurane | 2.0 % | 1.25 % | Pure air | 12 | 6.5 (4.0) |
| Standard isoflurane + ventilation | 3.5 % | 1.75 % | 3:1 | 5 | 9.2 (3.3) |
| Medetomidine s.c. | 3.5 % | 0.15 mg/kg bolus;  0.30 mg/kg/h infusion | 3:1 | 11 | 17.1 (6.5) |

Supplementary table 2. Median and interquartile ranges (IQR) of respiration and heart rates for the different anesthesia protocols and mouse strains. Values apply to C57Bl/6J mice only. The distributions of the respiration and heart rates were different for the four protocols, both during baseline and during CO2. Post-hoc analysis indicated that:

- the medetomidine respiration rate was higher than the medium isoflurane (adj. p = 0.001) and the isoflurane + ventilation protocol (adj. p = 0.007) during baseline
- the medetomidine respiration rate was higher than the medium isoflurane (adj. p < 0.001) and the low isoflurane protocol (adj. p = 0.019) during CO2
- the medetomidine heart rate was higher than the medium isoflurane (adj. p = 0.009) and the low isoflurane protocol (adj. p = 0.026) during baseline
- the medetomidine respiration rate was higher than the medium isoflurane (adj. p = 0.007) and the low isoflurane protocol (adj. p = 0.047) during CO2

Also note the paradoxical respiration rate decrease during CO2 in the low isoflurane protocol in C57Bl/6J mice.

| **Anesthesia**  **protocol** | **Mouse strain** | **Respiration rate baseline** | | **Respiration rate during CO2** | | **Heart rate baseline** | | **Heart rate during CO2** | |
| --- | --- | --- | --- | --- | --- | --- | --- | --- | --- |
|  |  | Median | IQR | Median | IQR | Median | IQR | Median | IQR |
| **Medium isoflurane** | C57BL/6J | 83 | 16 | 89 | 31 | 500 | 138 | 550 | 119 |
| **Low isoflurane** | C57BL/6J | 151 | 20 | 141 | 13 | 424 | 61 | 429 | 35 |
|  | B6C3 | 154 | 50 | 155 | 32 | 492 | 101 | 452 | 53 |
| **Medium isoflurane + ventilation** | C57BL/6J | 91 | 32 | 149 | 53 | 379 | 148 | 409 | 56 |
|  | B6C3 | 105 | 54 | 157 | 22 | 474 | 46 | 449 | 73 |
| **Medetomidine** | C57BL/6J | 173 | 29 | 209 | 34 | 289 | 50 | 335 | 88 |

Supplementary table 3. Median and interquartile ranges (IQR) of Cerebral Blood Flow (CBF), Cerebrovascular Reactivity (CVR) and Time to Half Peak (TTHP) values for the different anesthesia protocols used. Values apply to C57Bl/6J mice only.

| **Anesthesia**  **protocol** |  | **Baseline CBF**  **(mL/100 g/min)** | | **CBF during CO2 challenge**  **(mL/100 g/min)** | | **CVR (%)** | | **TTHP (sec)** | |
| --- | --- | --- | --- | --- | --- | --- | --- | --- | --- |
|  |  | Median | IQR | Median | IQR | Median | IQR | Median | IQR |
| **Medium isoflurane** | Cortex | 173.1 | 61.7 | 183.0 | 56.7 | 0.56 | 7.35 | 31.5 | 22.75 |
|  | Full Brain | 157.7 | 41.7 | 161.3 | 41.6 | 0.54 | 3.1 | 38.5 | 36.8 |
| **Low isoflurane** | Cortex | 143.0 | 38.3 | 164.1 | 28.1 | 12.7 | 9.3 | 31.5 | 7.0 |
|  | Full Brain | 138.9 | 26.5 | 151.5 | 24.5 | 10.0 | 7.1 | 31.5 | 7.0 |
| **Medium isoflurane + ventilation** | Cortex | 136.1 | 14.2 | 157.1 | 14.5 | 12.2 | 23.5 | 24.5 | 12.3 |
|  | Full Brain | 131.7 | 10.0 | 141.8 | 7.8 | 9.6 | 15.1 | 24.5 | 24.5 |
| **Medetomidine** | Cortex | 99.7 | 37.6 | 153.1 | 43.6 | 43.4 | 31.6 | 66.5 | 59.5 |
|  | Full Brain | 84.4 | 31.4 | 122.3 | 39.8 | 51.7 | 25.7 | 59.5 | 29.8 |

Supplementary table 4. Pros and cons of the anesthesia protocols tested.

|  | **Pros** | **Cons** |
| --- | --- | --- |
| **Medium isoflurane** | - Easiest protocol  - Short imaging session | - No CVR experiment possible  - Respiratory depression |
| **Low isoflurane** | - Accessible protocol for any user  - CVR measurable | - Slightly longer induction time of anesthesia  - Quick transfer to MRI animal bed after induction needed |
| **Medium isoflurane + mechanical ventilation** | - Good gas exchange in the lungs  - CVR measurable | - Technical skills required for the intubation procedure  - Dedicated equipment needed (ventilation pump)  - Longer preparation time (intubation procedure)  - Choice to make: either extra noise due to counter-breathing or extra animal discomfort due to paralysis |
| **Medetomidine** | - High CVR measurable | - Not suitable for every mouse strain  - Longer preparation time (injection line, injection minipump)  - Narrow time window for CVR measurements; measuring too early results in an unstable baseline; too late and the mouse is awake  - Cardiac depression |

**Supplementary figures**


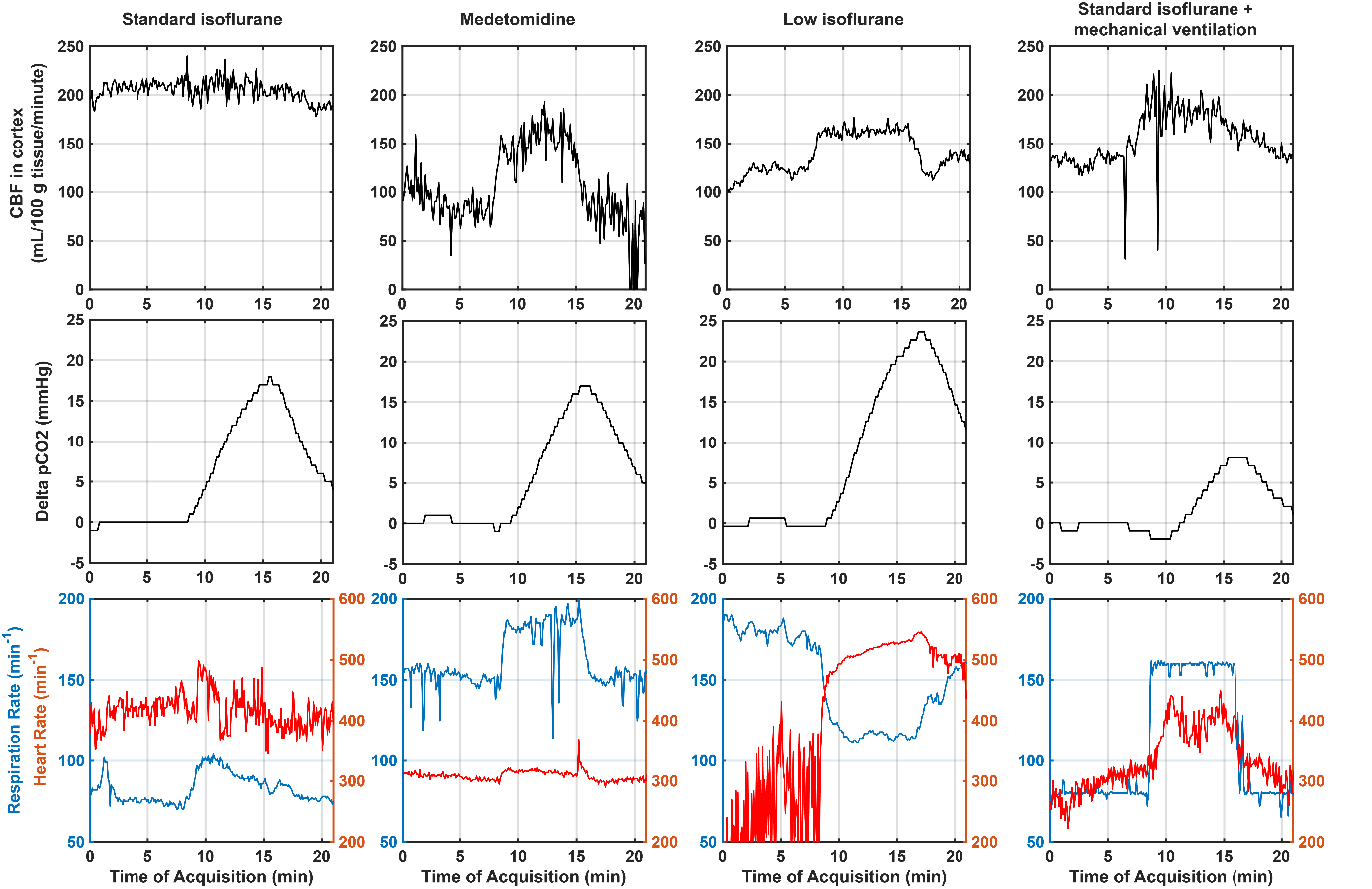


**Supplementary figure 1. Examples of physiological time-profiles.** Note the incorrect sampling of the heart rate in the first half of the time-profile of the low isoflurane protocol. Also note the doubling of the measured respiration rate (ventilated at 80bpm) in the ventilation protocol, due to extra breaths/movements that the mouse exhibited in sync with the ventilation protocol.

**
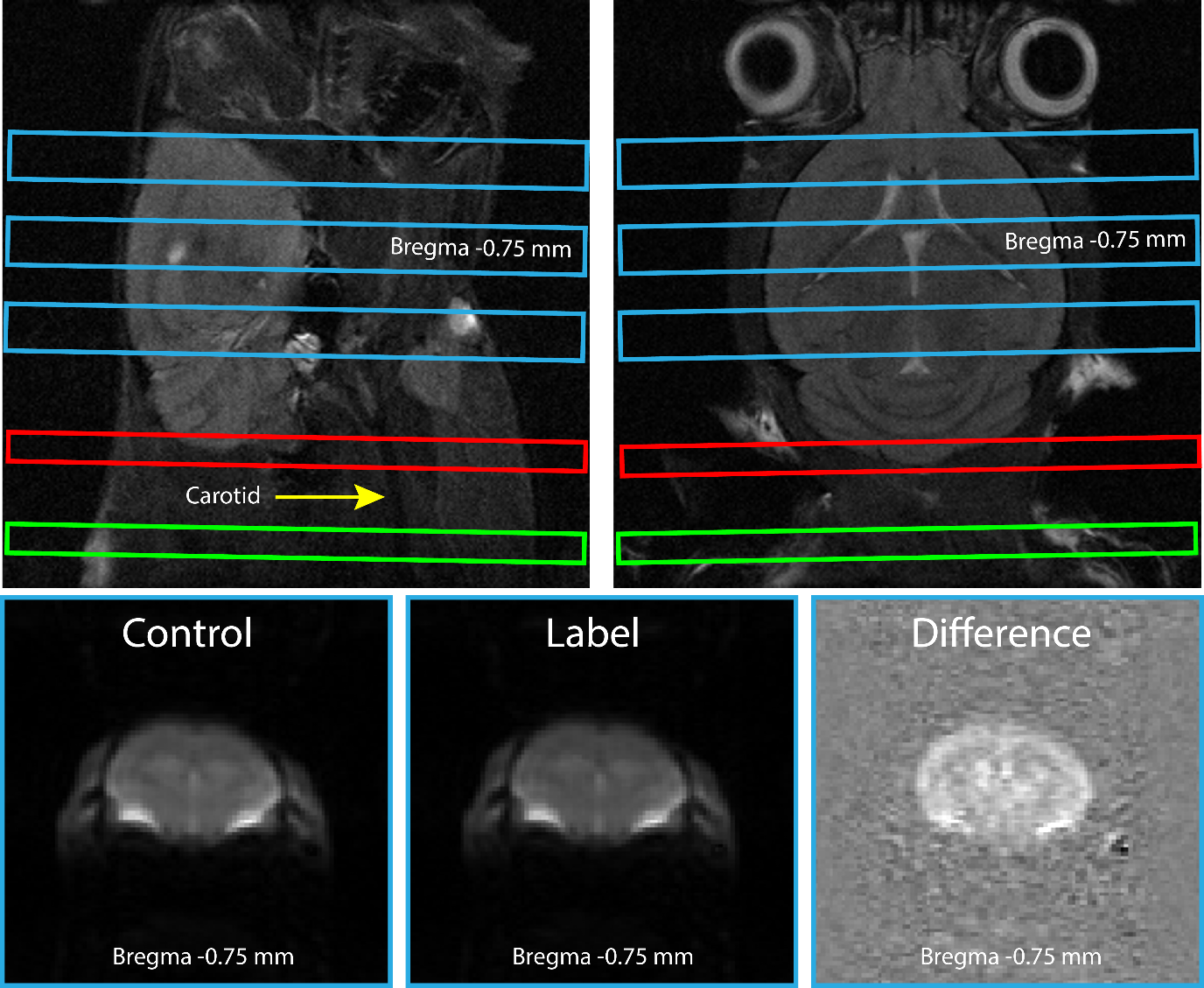
**

**Supplementary figure 2. Example of the ASL spatial planning and resulting ASL images.** The planning is shown on sagittal (upper left) and coronal (upper right) T2-weighted RARE images. The label slice is depicted in green, the inversion efficiency imaging slice in red and the imaging slices in blue. The yellow arrow indicates the location of one of the two carotid arteries. Shown below is one pair of ASL images (both label and control), corresponding to the middle slice shown on the planning. Also shown is the relative difference of the two (subtraction of the label from the control, divided by the control).

**
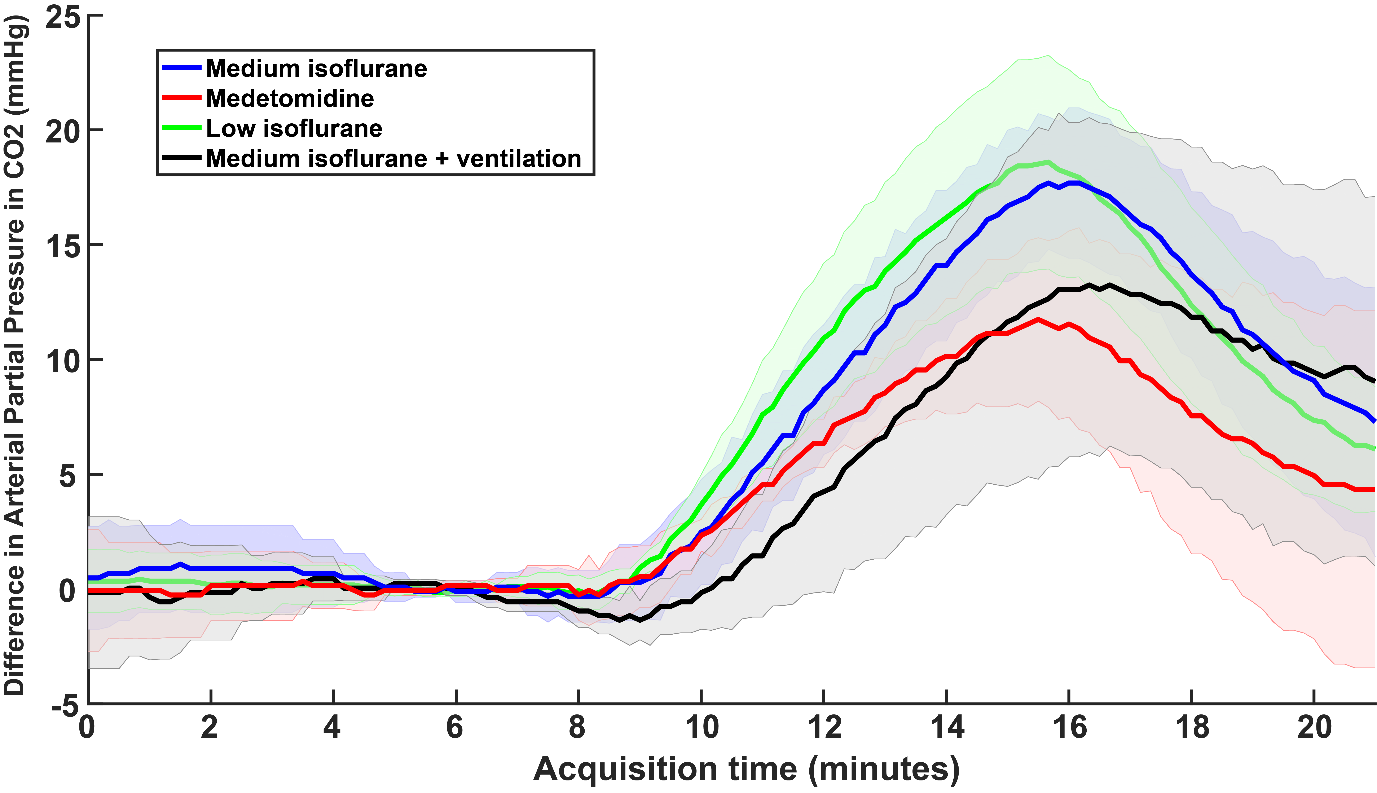
**

**Supplementary figure 3. The transcutaneous pCO2 profiles measured during the pCASL scans in the C57BL/6J mice.** The profiles are grouped per anesthesia protocol (mean ± SD). No significant differences were found between the groups, H(3) = 6.167; p = 0.104. Note that it takes around 2 minutes before the arterial CO2 has diffused to the skin (CO2 was administered from minute 7-14). Also to be mentioned is that 7 out of the 11 medetomidine profiles have not been captured due to a technical error.


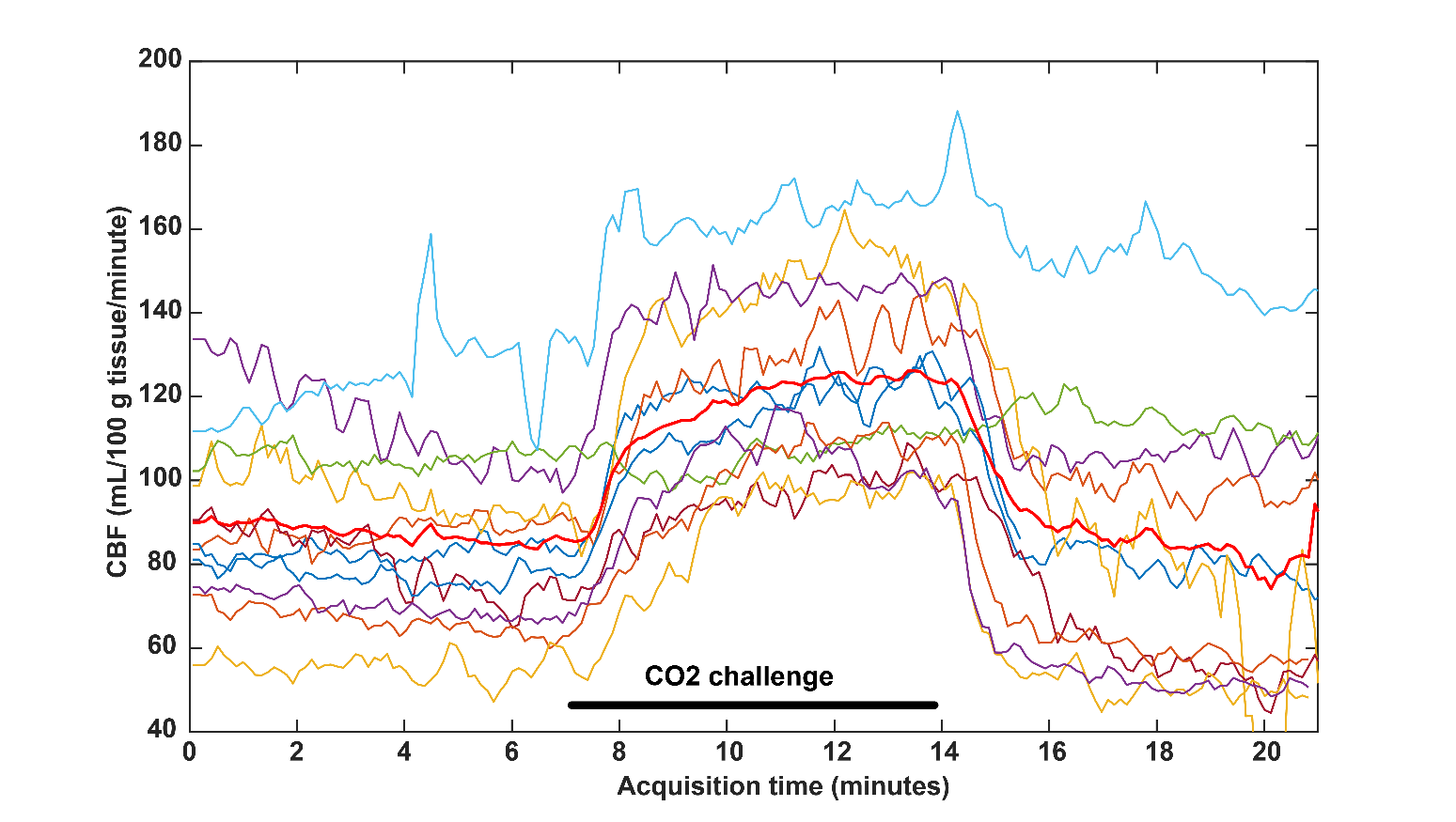


**Supplementary figure 4. Individual time-profiles of the animals in the medetomidine group.** Every line represents the results of the full brain ROI from one mouse. The red line indicates the group average. Note that some of the individual profiles are not stable yet in the first minutes of the scan.
